# Behavioral examination of the role of the primary visual cortex in the perceived size representation

**DOI:** 10.1038/s41598-023-48632-1

**Published:** 2023-11-30

**Authors:** Sang-Ah Yoo, Sung Jun Joo

**Affiliations:** Department of Psychology, Pusan National University, Busan, Republic of Korea

**Keywords:** Psychology, Human behaviour

## Abstract

Previous research has shown that neural activity in the primary visual cortex (V1) and V1 surface area may be linked with subjective experience of size illusions. Here, we behaviorally measured the hallway illusion with experimental manipulations as a proxy of V1’s influence on size perception. We first tested whether the hallway illusion can persist without further recurrent processing by using backward masking. Next, we examined relations among the hallway illusion magnitude and other perceptual measures that have been suggested to be correlated with V1 surface area. In Experiment 1, the magnitude of the hallway illusion was not affected by the stimulus duration and visual masking when the hallway context was previewed (i.e., complex depth information is already processed). It suggests that V1 activity could support the size illusion to some extent even when recurrent processing between V1 and higher areas is disturbed. In Experiment 2, the hallway illusion magnitude was correlated with the Vernier acuity threshold, but not with physical size discriminability. Our results provide converging evidence with the previous findings in that neural activity in V1 may contribute to size illusions and that V1 surface area is not the sole factor that mediates size perception and visual precision.

## Introduction

The primary visual cortex (V1) has a retinotopic organization^1–4^, which leads to a prediction that V1 activity should reflect the spatial extent of the visual stimulus in the retinal image. Nevertheless, representing the stimulus size is not solely dependent on its retinal size but the surrounding visual contexts where it is located also matter. Studies often use visual illusions to investigate the effects of visual contexts (e.g., depth cues) on size perception. For example, in the Ponzo illusion^5^, the size of the two physically identical stimuli looks different from each other if they are located in the depth-inducing background. A previous study using the hallway illusion, a modified version of the Ponzo illusion, demonstrated that activity in human V1 changed depending on perceived size, rather than the retinal angular size^6^. This finding is consonant with the earlier electrophysiological results which implied that the computational mechanism for size-distance scaling is presented as early as in V1^7,8^, and further supported by a series of subsequent studies (Ponzo illusion^9–13^, other size illusions^12,14–17^).

One major source of V1’s sensitivity to perceived size in the Ponzo-like context could be feedback signals from higher visual areas^6,9,11,18,19^ (see also^17,20–23^ for compatible findings). Analysis on the depth information in a given visual scene is likely to occur beyond V1 where complex visual information can be processed and integrated^7,24–26^, and then, the outcomes of this analysis are fed back into V1, modulating the response of V1 neurons. In the similar hallway context used in Murray et al.^6^, the spatial distribution of V1 activity was shifted along the eccentricity dimension depending on perceived size, and importantly, narrowing the attentional focus with a demanding fixation task reduced this effect^9^. With regard to the feedback account, this might have been due to the reduction in feedback signals from higher-order visual areas that process the depth information in the surrounding contexts. The electrophysiological counterpart showed that the receptive fields (RFs) of V1 neurons in monkeys shifted in response to perceived size^11^. Nevertheless, studies have also shown that the effects of the Ponzo-like illusions can emerge very quickly at the behavioral level^27–30^, although when the illusion strength reaches its maximum is controversial. The rapid emergence of the Ponzo-like illusions implies that V1 activity could support the illusions to some extent during initial feedforward sweep of visual processing.

Interestingly, the anatomical structure of V1 also seems to be associated with the representation of perceived size. Studies demonstrated that the magnitudes of size illusions, or contextual effects, were reduced as functionally defined V1 surface area increased^12,15,31^. It has been suggested that the spatial scale of lateral interaction plays an important role in these effects^12,32,33^: the larger V1 surface area, the lateral spread of dendrites covers a smaller area in retinotopic space and thus, the spatial extent of lateral interaction becomes weaker. Furthermore, V1’s population receptive field (pRF) size tends to be smaller for a larger V1^34^, predicting the same outcome. On the other hand, V1 surface area is positively correlated with orientation discrimination^35^, spatial position discrimination sensitivity^36^, and cortical magnification at an eccentricity is also a predictor of Vernier acuity^37^. Hence, one may speculate that V1 surface area would be related to visual precision and further mediate the ability to precisely discriminate size in the absence of (illusory) surrounding context. This sensitivity to fine visual details could also influence subjective percept of size illusions, if it is regarded as a failure of precise size discrimination. The previous studies, however, reported mixed findings on the link between the Ebbinghaus size illusion and physical size discrimination^31,38^, leaving the underlying mechanism of the two size perception processes elusive. Since different size illusions are likely to be supported by different processes^12,33,39–41^, the results from the Ebbinghaus illusion could be illusion-specific.

In our research, we aimed to further assess the behavioral outcomes that may be derived from V1’s roles in perceived size representation. First, despite the importance of feedback in the Ponzo-like illusions, it has not been examined whether recurrent processing during stimulus presentation is still necessary. For instance, the initial feedforward sweep of stimulus processing may be sufficient for the hallway illusion when feedback signals are already in place. If recurrent processing between early and higher visual areas is not necessary, the illusion magnitude would not be affected by backward visual masking that disturbs recurrent processing. Secondly, we measured correlations among the magnitude of the hallway illusion, physical size discrimination, and Vernier acuity thresholds. With this, we tested whether perceived size representation and perceptual acuity are intertwined with each other at the behavioral level.

## Experiment 1

### Materials and methods

#### Participants

Six naive graduate and undergraduate students of Pusan National University (ages 20–26 years, 3 males) participated in Experiment 1. They had normal or corrected-to-normal visual acuity, and normal color vision. Written informed consent was obtained from all participants and they received course credits for their participation. All procedures were approved by the Departmental Review Board of Pusan National University and were performed in accordance with the guidelines of the Declaration of Helsinki.

#### Apparatus and stimuli

Experiment was conducted in a dark room. Participants sat 70 cm from a BENQ LCD monitor (XL2740, 27-in., 1920 × 1080, 120 Hz) and their heads were stabilized on a head-and-chin rest. We created the stimuli and controlled the experiment using MATLAB (The MathWorks Corp.) and the Psychophysics Toolbox^42,43^. In addition, we used the Radiance software package (Lawrence Berkeley National Laboratory) to render the three-dimensional (3D) images of the spheres and hallway as in Murray et al.^6^.

Two types of stimuli were used to measure participants’ size judgment performance in different visual contexts. First, in the hallway illusion task, a rendered 3D image of a hallway was presented as a background and two spheres were placed at bottom-left and top-right of the background, giving the impression that the bottom-left sphere is closer to a participant than the other. We will call the bottom-left and top-right spheres the “front” and “back” spheres, respectively. While the size of the back sphere was kept constant (6.28° (degrees of visual angle) in diameter), the size of the front sphere could be one of the following: 6.96, 7.15, 7.25, 7.35, 7.44, 7.54, and 7.73°. Since Murray et al. reported that their participants perceived the back sphere to be at least 17% larger than the front sphere when their size was physically the same, we had 7.35° (6.28° × 1.17) as the median of the possible front sphere sizes.

Second, for the size judgment task without the hallway background, two white circles were presented on the background which was a scrambled version of the hallway image. We divided the original hallway image into 16 × 12 blocks and randomly shuffled them to make the image visually unrecognizable but preserving the pixel values. The scrambled images were also used for backward masking in the hallway illusion task. The size of the top circle was 6.28° in diameter and the size of the bottom circle was selected among 5.89, 6.09, 6.19, 6.28, 6.38, 6.48, and 6.67°.

#### Task and procedure

**Figure 1:**
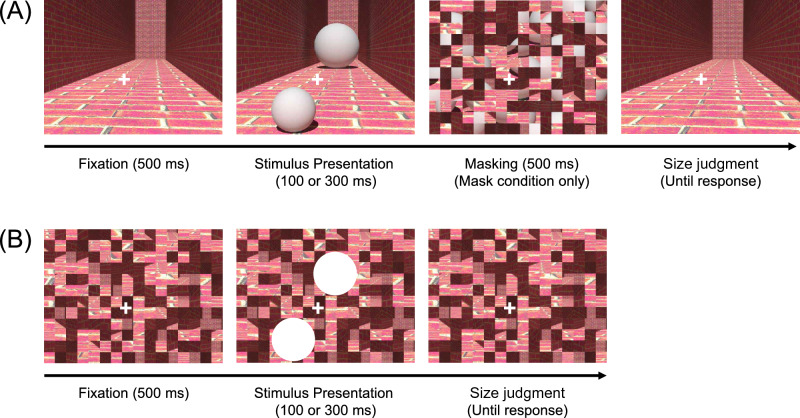
The procedures of (**A**) the hallway illusion task and (**B**) the size judgment task without the hallway background in Experiment 1. Participants reported which sphere or circle looked larger than the other.

Participants took part in the different tasks and conditions in random order. At the outset of the hallway illusion task, the hallway image and the fixation point were simultaneously presented for 500 ms (Fig. 1A). Participants were asked to keep looking at the fixation point throughout the task. The fixation point was presented halfway from each sphere, not at the exact center of the display. The distance from the fixation point to the center of a sphere was 5.35°. Subsequently, the two spheres were presented for either 100 or 300 ms and then, they could be masked for 500 ms or not, depending on the experimental conditions (mask vs. no-mask). The effects of different stimulus presentation durations and the presence of the mask were tested in separate experimental blocks. After masking or the mere disappearance of the two spheres, the hallway image was presented again. Participants were asked to report which sphere looked bigger by pressing preassigned keys and the hallway image remained on the screen until they responded. Each participant completed 840 trials in total (two stimulus presentation durations × presence/absence of the mask × seven front sphere sizes × 30 repetitions).

Participants also performed the size judgment task without the hallway background to test whether they could judge the size of stimuli correctly if there was no illusory context (Fig. 1B). The procedure of this task was the same as that of the hallway illusion task, except that there was no masking. Two white circles were presented at the locations where the spheres in the hallway illusion task were presented. Participants indicated which circle looked bigger than the other. This task consisted of 420 trials (two stimulus presentation durations × seven bottom circle’s sizes, 30 repetitions).

#### Data analysis

We measured points of subjective equality (PSEs) where the front sphere/bottom circle was equally likely to be judged as larger or smaller than the back sphere/top circle to quantify size judgment performance with or without the hallway background. A normal cumulative distribution function was used as a model of the psychometric function, and it was fitted to the individual participants’ data using the maximum likelihood method. We computed the size judgment errors (°) by subtracting the standard stimulus size from PSEs.

### Results

**Figure 2:**
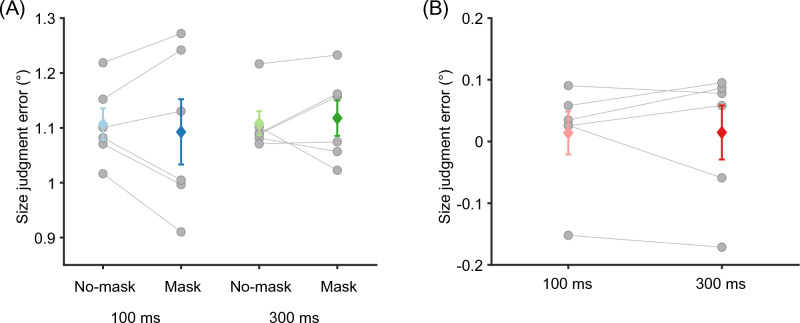
The results of Experiment 1. (**A**) The size judgment error measured in the hallway context was not significantly affected by stimulus presentation durations and mask conditions. Positive size judgment error indicates the hallway illusion. (**B**) The size judgment error when the hallway background was absent was not significantly different from zero and not affected by stimulus presentation duration. Colored diamonds indicate the mean of the data and error bars indicate the standard error of the mean (SEM). Each gray circle represents one participant.

First, we examined whether the hallway illusion was induced when the two spheres were embedded in the hallway background. When the two spheres were presented for 100 ms, the mean size judgment error was 1.09° ± 0.06° (SEM) and 1.11° ± 0.03° with and without masking, respectively (Fig. 2A). When the presentation duration was extended to 300 ms, the mean size judgment error was 1.12° ± 0.03° and 1.11° ± 0.02° with and without masking. The size judgment errors in all conditions were significantly greater than zero (all *p*s < 0.001), meaning that our participants experienced the hallway illusion. Neither the presentation duration nor the presence of masking influenced the size judgment error (presentation duration: *F*(1, 5) = 0.187, *p* = 0.683, masking: *F*(1, 5) = 0.008, *p* = 0.930), and their interaction did not have an impact on it, either (*F*(1, 5) = 0.886, *p* = 0.390). Most importantly, there was no significant difference in the size judgment error between the two extreme conditions (100 ms presentation with masking vs. 300 ms presentation without masking: *t*(5) = − 0.310, *p* = 0.769). This result suggests that perceived size of task-relevant stimuli can be represented rapidly even when further recurrent processing is disturbed.

In the size judgment task without the hallway background, the mean size judgment error was 0.01° ± 0.03° and 0.01° ± 0.04° when the two target circles were presented for 100 ms and 300 ms, respectively (Fig. 2B). For both presentation durations, the mean bias was not significantly different from zero (100 ms: *t*(5) = 0.399, *p* = 0.706, 300 ms: *t*(5) = 0.334, *p* = 0.752), indicating that our participants could precisely judge the size of target stimuli when there was no illusory context.

## Experiment 2

### Materials and methods

#### Participants

Fifty naive undergraduate students of Pusan National University (ages 19–31, 13 males) participated in Experiment 2 to fulfill a course requirement. They had normal or corrected-to-normal visual acuity, and normal color vision. Written informed consent was obtained from all participants and they received course credits for their participation. All procedures were approved by the Departmental Review Board of Pusan National University and were performed in accordance with the guidelines of the Declaration of Helsinki.

#### Apparatus and stimuli

We used the same apparatus as in Experiment 1, except that the viewing distance was 55 cm for the hallway illusion and physical size discrimination tasks, and it was 27.5 cm for the Vernier acuity task to reduce pixel density as the task requires finer-grained visual analysis.

The stimuli for the hallway illusion task were identical to those in Experiment 1. However, the stimulus size had been modified after changing the viewing distance. The size of the back sphere subtended 7.75° of visual angle and the size of the front sphere could be one of the following: 8.58, 8.82, 8.94, 9.06, 9.18, 9.30, and 9.54°. For the physical size discrimination task, the two white circles were presented on the uniform gray background. The size of the standard circle was 7.75° as in the hallway illusion task and the size of the test circle was adjusted using the QUEST adaptive procedure^44^ (see *Task and Procedure*). Lastly, for the Vernier acuity task, two 1°-long, white vertical lines were presented one above the other with 10' spatial separation on the uniform gray background. The offset between the two lines was also adaptively adjusted via QUEST.

#### Task and procedure

Participants performed the three different tasks in random order. The procedure of the hallway illusion task was the same as in Experiment 1, except that the two spheres were always presented for 100 ms with subsequent visual masking. The distance from the fixation point to the center of each sphere was 6.58° due to the change in the viewing distance. Each participant completed 210 trials in total (seven front sphere sizes × 30 repetitions).

The physical size discrimination task was used to measure the participant’s physical size discrimination ability. This task was similar to the one used in Experiment 1 (i.e., size judgment task without the hallway background) but some modifications had been made. First, the size of the test circle changed using the two randomly interleaved QUEST staircases, not by the method of constant stimuli. By doing this, we measured size discrimination thresholds rather than PSEs. Second, the locations of the standard and test circles were randomized every trial. For example, the standard circle was not always presented at the top-right of the display, but it could be also presented on the bottom-left of the display where the test circle was always presented in Experiment 1. Third, the circles were always presented for 100 ms and then masked with alternating checkerboard patterns for 500 ms. Lastly, the color of the fixation point changed depending on participants’ response accuracy—green for correct and red for incorrect responses. There were 10 practice trials and the main task consisted of 240 trials (four blocks × two staircases × 30 trials). Participants also performed six additional dummy trials in the beginning of each block to stabilize the judgments.

**Figure 3:**
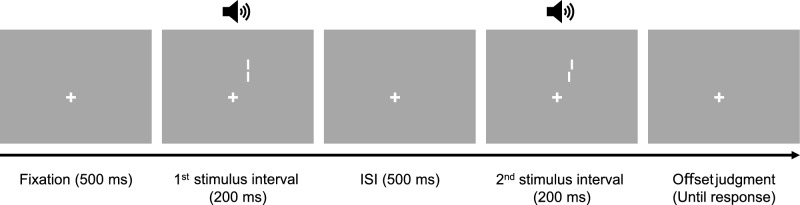
The procedure of the Vernier acuity task in Experiment 2. Participants indicated which stimulus interval contained the vertically misaligned lines.

Figure 3 illustrates the procedure of the Vernier acuity task. In the beginning of the task, a fixation point was presented for 500 ms. The fixation point remained on the screen until the end of the task and participants were asked to keep looking at this throughout the task. The two lines were presented simultaneously for 200 ms in the two successive intervals with 500 ms ISI. Each stimulus interval was synchronized with a beep sound to be clearly distinguished. The lines were equally likely presented at the top-right or bottom-left of the display (the location changed every block), matching the eccentricity of the back sphere/top circle or the front sphere/bottom circle in the other tasks. After stimulus presentation, participants indicated the stimulus interval where the two lines were not vertically aligned by pressing preassigned keys. Visual feedback for each response was given by changing the color of the fixation point. The offset between the lines was adjusted using the two randomly interleaved QUEST staircases. Each participant completed the same number of trials as in the physical size discrimination task.

#### Data analysis

In the hallway illusion task, we computed the size judgment error by subtracting the standard stimulus size from PSEs as in Experiment 1. Six participants’ size judgment errors were excluded from the analysis because these participants did not properly perform the task (e.g., responded that a certain sphere looked bigger than the other in all or most of the trials, or made random responses). In this case, PSEs were inflated and sometimes they were even greater than the biggest test circle’s size (9.54°), resulting in unreliable outcomes. In the physical size discrimination and Vernier acuity tasks, we estimated each participant’s thresholds by averaging the last thresholds of individual QUEST staircases. Furthermore, in all the tasks, participants’ data fell outside three standard deviations of the mean were excluded from the analysis (one data point from each task). Consequently, 43 participants’ data from the hallway illusion task, 49 from the physical size discrimination task, and 49 from the Vernier acuity task were included in the analysis.

### Results

When the size of the spheres was judged in the hallway context, the mean size judgement error was 1.31° ± 0.04° (SEM) and it was significantly greater than zero, indicating the manifestation of the size illusion (*t*(42) = 32.889, *p* = 1.319 × 10^–31^). The mean physical size discrimination threshold was 0.37° ± 0.02° and the mean Vernier acuity threshold was 5.05' ± 0.21'.

**Figure 4:**
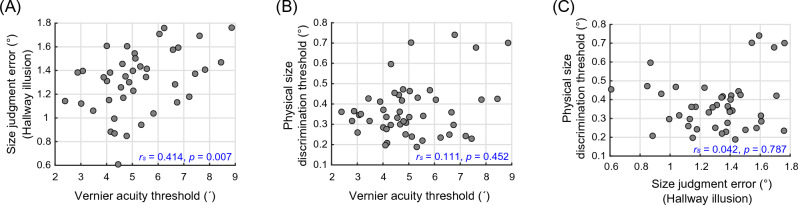
The results of Experiment 2. Each scatter plot illustrates the relation between the measures of two tasks: (**A**) The Vernier acuity threshold and size judgment error (hallway illusion), (**B**) the Vernier acuity and physical size discrimination thresholds, and (**C**) the size judgment error and physical size discrimination threshold. Spearman’s rank correlation (r_s_) was computed to quantify the relations between different perceptual capabilities.

As the main interest of Experiment 2, we analyzed the relations among different tasks to examine the existence of their common underlying mechanism. We report Spearman’s rank correlation which is more resistant to outliers than Pearson’s correlation. The Vernier acuity threshold was positively correlated with the size judgment error (hallway illusion) (*r*_*s*_(41) = 0.414, *p* = 0.007, Fig. 4A), meaning that participants who are less sensitive to fine-grained spatial information tended to experience a stronger hallway illusion. Along with the previous findings, the current result implies the association between visual acuity and size illusions, potentially mediated by V1 surface area^31,37^.

On the other hand, the Vernier acuity and physical size discrimination thresholds were not significantly correlated (*r*_*s*_(47) = 0.111, *p* = 0.452, Fig. 4B) and importantly, no significant correlation was observed between the size judgment error (hallway illusion) and physical size discrimination threshold (*r*_*s*_(42) = 0.042, *p* = 0.787, Fig. 4C). This null result may indicate that subjective experience of the hallway illusion and discriminating physical stimulus size are likely to be supported by different factors. This result is also consistent with the previous one which was found in the Ebbinghaus illusion^31^, although the two illusions are thought to be mediated by different mechanisms^12,33,39–41^.

## Discussion

In the present research, we examined behavioral outcomes that may associated with the role of V1 in the representation of perceived size. First, we studied the temporal aspect of the perceived size representation in the hallway illusion. We assessed whether the hallway illusion can be induced rapidly without further recurrent processing if processing of complex depth information precedes. We reasoned that V1 would support the rapid emergence of the hallway illusion during the initial feedforward sweep if feedback information is already given. Secondly, we investigated whether the magnitude of the hallway illusion, physical size discrimination and Vernier acuity thresholds were related to each other, presumably supported by V1 surface area^12,37^.

In Experiment 1, we did not observe significant changes in the illusion magnitude across the different presentation durations (100 vs. 300 ms) and mask conditions. This result suggests that perceived stimulus size can be represented rapidly without recurrent processing during stimulus presentation. Note that the hallway context was always presented for 500 ms before the onset of task-relevant stimuli (Fig. 1A). During this time, the feedback signals that relay complex depth information in the hallway background might have already reached V1, and the initial feedforward sweep of task-relevant stimulus processing would be sufficient for the hallway illusion to occur. It implies that V1 is not a passive recipient of feedback signals but it may flexibly combine feedforward retinal and feedback signals, partly responsible for forming an integrated representation of the perceived visual input. Our finding is consistent with the rapid manifestation of the Ponzo-like illusions^27–30^, but it appears that the strength of the illusion reached its peak earlier than 100 ms, not showing a further increment when additional processing time was given. It was equivocal whether the illusion strength continues to develop over the next hundreds of milliseconds. The heterogeneity of the findings may arise due to different measurements across studies and the hallway background in our experiment which contained richer depth cues as the illusion magnitude is correlated with the number of available depth cues^6,45^.

In Experiment 2, we examined whether the hallway illusion magnitude, Vernier acuity, and physical size discrimination thresholds are correlated with each other. If V1 surface area underlies size representation and also visual precision, there could be certain relations among these three tasks. As results, illusion magnitude was positively correlated with Vernier acuity threshold, but the correlations among the other tasks were not significant. Since previous studies reported that V1 surface area mediates Vernier acuity^37^ and the Ebbinghaus illusion^31^, the correlation between the Vernier acuity threshold and hallway illusion magnitude appears to be valid. It also suggests a possibility that V1 surface area may be associated with various size illusions. Nevertheless, care should be exercised in interpreting the present results. We cannot be sure about the relation between V1 surface area and the hallway illusion because we did not directly measure it. In addition, the mechanisms for the hallway and Ebbinghaus illusions are assumed to differ^12,33,39–41^, thus, it must be examined if other confounding factors had influenced the results.

There have been mixed reports regarding the correlation between physical size discrimination and the Ebbinghaus illusion magnitude^31,38^. These discrepant results might be due to different ways of stimulus presentation. While our and Schwarzkopf et al.^31^ simultaneously presented stimuli, Chen et al.^38^ successively presented stimuli with a relatively long temporal gap (800–1200 ms) between them, which requires storing the size information in memory^46^. Discrimination thresholds were much smaller in simultaneous presentation compared to non-simultaneous presentation, suggesting that individual differences in the sensitivity of physical size discrimination may be more pronounced in successive presentation and compressed in simultaneous presentation^47^.

Although V1 surface area is correlated with perceptual tasks that require high-resolution visual representation, such as Vernier acuity^37^ and orientation discrimination^35^, physical size discrimination seems to be mediated by a different mechanism. For instance, Moutsiana et al.^15^ demonstrated that basic size perception in the absence of illusory contexts is associated with pRF size. Small pRFs enable fine-grained representation of visual inputs so it may support physical size discrimination. This claim, however, seemingly contradicts the finding that V1 surface area is negatively correlated with pRF size^34^, thus V1 surface area is indirectly linked with physical size discrimination. To resolve this issue, the authors disentangled the potential underlying factors for different size perception and suggested the dissociation between V1 surface area and pRF size. This study implies the existence of multiple candidates for different size perception. Perhaps, they may reside beyond V1. A blindsight patient whose V1 was surgically removed could report the size of after-images (generated by stimulating the blind field) following Emmert’s law^48^. Therefore, no single area but multiple cortical sites work together to support the representations of perceived size^49,50^. Future studies will have to explore the functional roles of different areas in size scaling and their connectivity.

As for the locus of size perception, a recent study suggests that the brain region with sufficiently large RFs, which pool visual inputs across different regions of the visual field would be the best candidate for size representation^51^. V1 neurons’ RFs are too small for this and V1 activity that appears to be associated with size representation is likely to be the result of feedback processing. While our research does not address where the size perception takes place, our results suggest that V1 is at least partly involved in size perception. Another issue worth considering is the operational definition of the term “size” in research. In many studies, including ours, task instructions simply ask participants to judge which stimulus looks bigger/smaller than the other without specifying what size means. “Size” is a loaded term so it can indicate the two-dimensional image size on the screen, or it can be interpreted as the object size in 3D, real-world situations where various depth cues are available. Thus, future studies should use more careful language for their participants and readers to clarify what they indeed measure.

In sum, our study shows that when feedback information is already in place, the hallway illusion emerges even though following recurrent processing is blocked. It suggests that feedforward V1 activity may contribute to perceived size representation without further recurrent processing between V1 and higher areas during stimulus presentation. Furthermore, the magnitude of the hallway illusion was correlated only with Vernier acuity threshold, but not with physical size discrimination threshold, supporting a previous finding using the Ebbinghaus illusion^31^. It implies that V1 surface area is not the unitary factor that mediates all the perceptual capabilities that necessitate fine-grained spatial judgments and that subjective experience on size illusions may not be based on the physical size discriminability.

## Data Availability

All data generated or analyzed during this study and the analysis codes are available in the public repository (https://osf.io/f9je3/).
